# Longitudinal mediation analysis with multilevel and latent growth models: a separable effects causal approach

**DOI:** 10.1186/s12874-024-02358-4

**Published:** 2024-10-25

**Authors:** Chiara Di Maria, Vanessa Didelez

**Affiliations:** 1 Department of Economics, Business and Statistics, University of Palermo, Viale delle Scienze, Building 13, Palermo, 90128 Italy; 2 Leibniz-Institut für Präventionsforschung und Epidemiologie - BIPS, Achterstraße 30, Bremen, 28359 Germany

**Keywords:** Longitudinal mediation analysis, Separable effects, Causal effects, Multilevel models, Latent growth models

## Abstract

**Background:**

Causal mediation analysis is widespread in applied medical research, especially in longitudinal settings. However, estimating natural mediational effects in such contexts is often difficult because of the presence of post-treatment confounding. Moreover, many models frequently used in applied research, like multilevel and latent growth models, present an additional difficulty, i.e. the presence of latent variables. In this paper, we propose a causal interpretation of these two classes of models based on a novel type of causal effects called separable, which overcome some of the issues of natural effects.

**Methods:**

We formally derive conditions for the identifiability of separable mediational effects and their analytical expressions based on the g-formula. We carry out a simulation study to investigate how moderate and severe model misspecification, as well as violation of the identfiability assumptions, affect estimates. We also present an application to real data.

**Results:**

The results show how model misspecification impacts the estimates of mediational effects, particularly in the case of severe misspecification, and that the bias worsens over time. The violation of assumptions affects separable effect estimates in a very different way for the mixed effect and the latent growth models.

**Conclusion:**

Our approach allows us to give multilevel and latent growth models an appealing causal interpretation based on separable effects. The simulation study shows that model misspecification can heavily impact effect estimates, highlighting the importance of careful model choice.

## Introduction

In recent years mediation analysis has increasingly been used with the aim to investigate causal mechanisms underlying phenomena of interest. In mediational settings, the effect of a treatment or an exposure on a certain response variable may be conveyed by a third variable called *mediator*. For example, physical activity may have a causal effect on blood pressure, at least in part mediated by body fat percentage. Given the dynamic nature of many phenomena, including the one in the example just provided, causal effects are generally not instantaneous and may need time to manifest. As a consequence, longitudinal data are particularly suited, if not required, to address mediation.

Many methods have been proposed to deal with longitudinal mediation analysis. Some of them fall under the SEM framework, such as cross-lagged panel models [1], latent growth models [2, 3] and latent difference score models [4]. Other models account for longitudinality by including mixed effects [5, 6], other else consider a dynamic process perspective, the so-called dynamic path analysis [7, 8]. Due to the fact that in epidemiology the interest often lies in time-to-event outcomes, several methods have been developed to address mediation in a survival setting: [9] adopt a semiparametric approach based on influence functions, [10] propose a generalisation of dynamic path analysis, while many authors [11–13] propose a mediational g-formula for estimating effects. Finally, there are also examples of continuous-time methods based on derivatives [14–16].

In this paper, we will focus on two models frequently used for longitudinal analysis and which share some similarities, i.e. multilevel (or mixed-effect) models [17, 18] and latent growth models (LGMs, [19]). Both models have been discussed from a causal perspective using the so-called *natural mediational effects* [20, 21]. However, the identifiability of these natural (in)direct causal effects, i.e. the ability to express them as a function of observed data, relies on the absence of post-treatment confounding. Bind et al. [5] addresses longitudinal mediation analysis using mixed-effect models, but only in settings without time-varying confounding, i.e. covariates which vary over time and whose presence violates classical assumptions required for non-parametric identification of natural effects [13]. Another limitation of natural mediational effects is the fact that they are defined in terms of nested counterfactuals [21, 22], i.e. counterfactuals depending on other counterfactuals. In particular, natural mediational effects are defined as contrasts of two counterfactual values of the response, which depend on counterfactual values of the mediator. In other words, natural effects entail conceptualizing a specific intervention on the mediator, which is not always straightforward or meaningful as it involves setting the treatment to two different values simultaneously. For example, [23] propose a causal interpretation of LGMs through natural effects, but their approach implies devising an intervention on mediators, which, in the case of LGMs, are latent variables. This can be rather counterintuitive and the practical relevance is limited.

An alternative approach to mediation analysis, called *separable (treatment) effects* was proposed by [24] (the name was coined by [25]), and recently applied to longitudinal mediation settings with a time-to-event outcome [26–28]. This approach relies on the assumption that the exposure or treatment can be separated into two (or more) components, one having a direct effect only on the mediator and the other one directly activating only the outcome. Some of its key advantages are that it does not require the notion of an intervention on the mediators, since only the components of the exposure are considered as separate interventional targets, and that causal effects can easily be interpreted in terms of modifications of these components. The separable effects approach overcomes some of the issues characterizing natural effects, since separable causal effects are defined without nested counterfactuals and their identifiability is obtained under assumptions that are in principle testable [29] as they do not involve cross-world independencies, unlike natural effects [30].

In this article, we propose a causal interpretation of generalised mixed-effect models and latent growth models in terms of separable effects. This enables us to provide explicit assumptions for endowing mediational effects with such an interpretation, and these assumptions can easily be interpreted from an interventional point of view [24, 31]. Furthermore, we prove that, in the linear case, formulas for the separable effects are consistent with those derived from path analysis.

## Methods

In this section, we first introduce separable mediational effects and then we discuss their application to multilevel and latent growth models. We also describe the simulation study carried out to evaluate how model misspecification affects the estimates of causal effects and show the results of the data analysis.

## Separable mediational effects

Causal mediation analysis has traditionally been addressed in the counterfactual framework. Denoting by *X*, *M* and *Y* the exposure, the mediator and the outcome, respectively, let *M*(*x*) and *Y*(*x*) be the value of the mediator and the response, respectively, if *X* were set to *x*, and let *Y*(*x*, *m*) denote the value of the response had *X* been set to *x* and *M* to *m*. Direct and indirect effects are usually defined as contrasts (the difference or the ratio) of nested counterfactual quantities [20, 21]: *Y*(*x*, *M*(*x*)) and $Y(x^{\prime} , M(x))$ for the direct effect, *Y*(*x*, *M*(*x*)) and $Y(x, M(x^{\prime} ))$ for the indirect one, with $Y(x, M(x^{\prime} ))$ denoting the value of the outcome had *X* been set to *x* and the mediator to the *natural* value it would have had if *X* were set to $x^{\prime}$, being $x \not = x^{\prime}$. For this reason, these effects are generally called *natural* [21].

The main problem of causal inference is how to express counterfactual quantities in terms of observed data, an issue known as *identifiability*. For natural effects to be identifiable from observational data, specific assumptions about confounding are required. Specifically, the exposure-mediator, exposure-outcome and mediator-outcome relationships should be unconfounded. Moreover, another condition is the so-called *cross-world independence assumption*. As its name suggests, this assumption involves counterfactuals referring to two different ‘worlds’ or scenarios, since it states that the counterfactual value of the outcome if *X* were set to *x* is independent of the mediator if *X* were set to a different value $x^{\prime}$, i.e. $Y(x,m) \perp \kern-.4cm \perp \,\, M(x^{\prime} )$.

The definition of natural mediational effects relies on nested counterfactuals, which require to think of setting the exposure to two different values at the same time, which may be quite counterintuitive, and to intervene on the mediator, a task sometimes even difficult to conceive. In addition, the cross-world independence assumption is untestable and can easily be violated in real settings, for example in the presence of an exposure-induced (or post-treatment) confounder [32], although [30] discuss other scenarios in which such an assumption can be violated. The separable effects approach overcomes these issues.

**Fig. 1:**
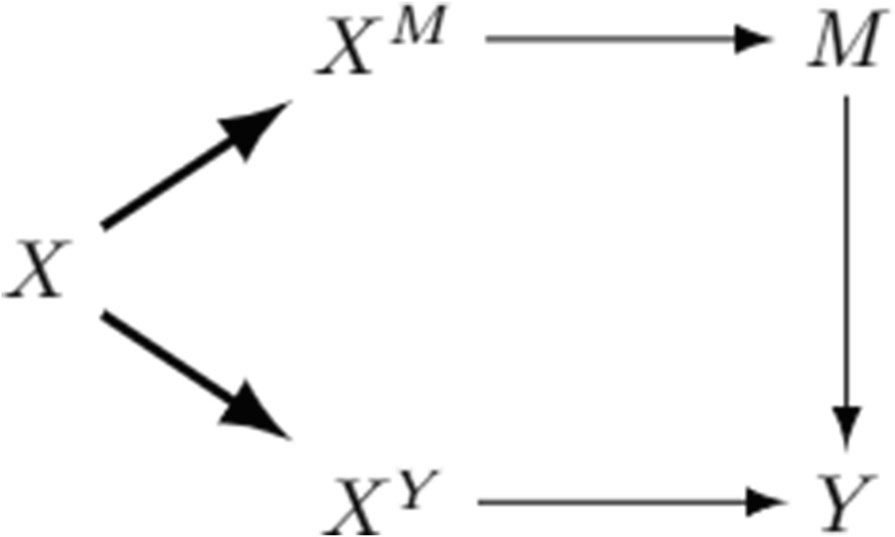
Separable effects via exposure decomposition

The basic idea underlying separable effects is to extend the model by including two additional variables, $X^M$ and $X^Y$, which can be thought of as two separate components of the exposure, the former influencing directly only the mediator, the latter only the outcome. This is graphically depicted in Fig. 1, since only an arrow emanates from $X^M$ and it goes into *M*, likewise there is only an arrow from $X^Y$ to *Y*. The introduction of these new variables allows for the definition of separable mediational effects as the contrasts of (single-world) counterfactuals involving only them, not the mediator. Although these variables are not observed, since we observe only the value of *X*, and in observational data it holds that $X \equiv X^Y \equiv X^M$, they turn out to be very useful to give insights into the mechanism linking *X* to *Y*. First, these additional variables allow us to disentangle the pathways through which the exposure effects propagate. As an example, suppose we are interested in the effect of breastfeeding on a child’s weight mediated by addictive behaviours like sugar craving: one can think of two distinct components of breastfeeding, one metabolic, affecting directly the child’s weight, and another neurological, affecting the addictive behaviour. Second, it is possible to conceive a future trial where the two *X* components are randomised independently. For example, the metabolic component is given by the nutritional compounds contained in the maternal milk, while the neurological component comes from the experience of breastfeeding, like the sense of closeness and attachment a child feels staying in the mother’s arms. In this setting, the two distinct components of *X* are intervening variables and, as we shall detail below, they allow us to define mediational effects without devising an intervention on the mediator, as required, instead, by natural effects. In the breastfeeding example, the metabolic component could be administered by giving children maternal milk through a baby bottle, the neurological component by reproducing a ‘breastfeeding situation’ without giving the milk. As another example, consider a study to investigate the effect of smoking cigarettes on the risk of myocardial infarction, mediated by hypertension. Robins and Richardson [24] argue that the natural effects proposed by Pearl involve thinking of intervening on subjects’ hypertensive status, but Pearl himself, to motivate the example, never mentions such an intervention, focusing instead on an intervention on two components of cigarettes, i.e. the nicotine and non-nicotine substances that they contain.

Third, the identification of separable mediational effects relies on weaker assumptions than those required by natural effects; in particular, they do not require any cross-world independence, since they are not defined in terms of nested counterfactuals. See [31] for a detailed discussion.

Our interest lies in longitudinal contexts, where the assumptions for identifiability of natural (in)direct effects are rarely plausible. This is due to the very likely presence of post-treatment confounding, i.e. confounders of *M* and *Y* affected by *X*. As already said, such variables violate the cross-world independence assumption, ruling out natural effects as possible estimands of longitudinal mediational effects. This is the reason why alternative definitions of causal direct and indirect effects are required. In this paper, we focus on separable effects, but other alternatives like interventional effects have been suggested [9, 11, 13].

We will consider a setting with a baseline binary exposure *X*, and a mediator *M* and a response *Y* measured over time for *T* different time occasions, where *M* is measured immediately before *Y*. The estimand of interest for each $t = 1, \dots , \, T$ is $\mathbb {E}[Y_t(X^Y = x, X^M = x^{\prime} )]$, that is, the expectation of the response at time *t* under a hypothetical intervention setting the components of *X* to two different values *x* and $x^{\prime}$. The mediational effects are indeed defined in terms of quantities of this kind, namely the *separable direct effect* (SDE) can be defined as a contrast

$$\begin{aligned} \mathbb {E}[Y_t (X^Y = x, X^M= x^{\prime} )] \qquad \text {vs}\qquad \mathbb {E}[Y_t (X^Y = x^{\prime} , X^M= x^{\prime} )] \end{aligned}$$

and the *separable indirect effect* (SIE) as a contrast of

$$\begin{aligned} \mathbb {E}[Y_t(X^Y = x, X^M= x)] \qquad \text {vs}\qquad \mathbb {E}[Y_t(X^Y = x, X^M= x^{\prime} )]. \end{aligned}$$

For these effects to be identifiable from data on $(X,\{M_t\},\{Y_t\})$, since $X^M$ and $X^Y$ are not observed, some assumptions are necessary. As in [26], we assume the following

- **Property P1** $$\mathbb {E}[Y(X^Y = x, \, X^M = x)] \equiv \mathbb {E}[Y(X=x)]$$.

Moreover, for any variable *W*, let $\overline{W}_t$ denote the history of $W_t$, i.e. the set of variables $W_k$ for $k \le t$.

- **A0** The treatment is randomised, implying that $\mathbb {E}[Y(X=x)] = \mathbb {E}[\, Y \, | \, X=x]$
- **A1** For each time *t*, the mediator $M_t$ is independent of the value of $X^Y$ conditional on its observed past, previous values of *Y* and $X^M$, $M_t \perp \kern-.4cm \perp X^Y \, | \, (\overline{M}_{t-1}, \overline{Y}_{t-1}, X^M)$
- **A2** For each time *t*, the response $Y_t$ is independent of the value of $X^M$ conditional on its observed past, previous values of *M* and $X^Y$, $Y_t \perp \kern-.4cm \perp X^M \, | \, (\overline{M}_{t}, \overline{Y}_{t-1}, X^Y)$

If these assumptions are satisfied, then, the quantity $\mathbb {E}[Y_t(X^Y = x, \, X^M= x^{\prime} )]$, where the components of *X* are set to two different values, is non-parametrically identifiable through the mediational g-formula [26]

1 $$\begin{aligned} \mathbb {E}[Y_t(X^Y = x,&X^M= x^{\prime} )] = \sum _{\overline{m}_t,\overline{y}_{t-1}} \mathbb {E}[Y_t \, | X=x, \, \overline{M}_t = \overline{m}_t, \overline{Y}_{t-1}= \overline{y}_{t-1}] \times \nonumber \\ &\prod _{k=1}^t P(\overline{M}_k = \overline{m}_k| X=x^{\prime }, \overline{M}_{k-1} = \overline{m}_{k-1}, \overline{Y}_{k-1} = \overline{Y}_{k-1} ) \times \nonumber \\ &P(\overline{Y}_{k-1} = \overline{y}_{k-1}| X=x, \overline{M}_{k-1} = \overline{m}_{k-1}, \overline{Y}_{k-2} = \overline{y}_{k-2}). \end{aligned}$$

**Fig. 2:**
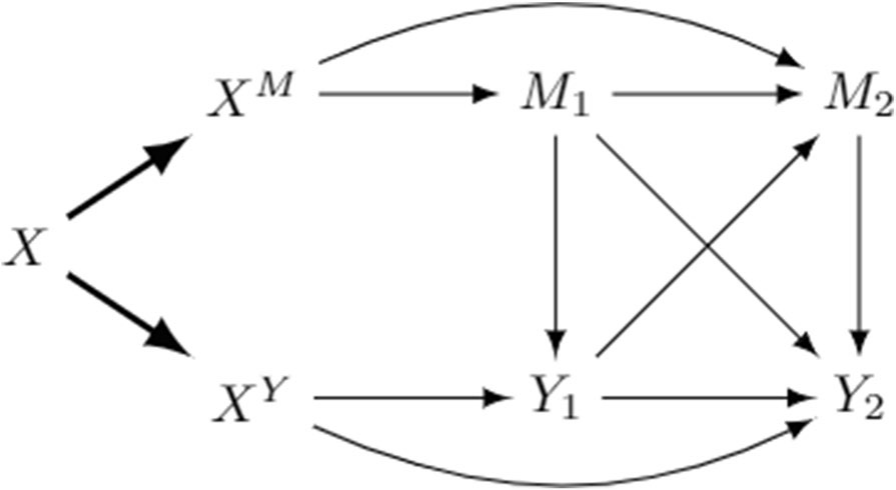
Longitudinal separable effect model with two time points

Assume that the causal structure is as in Fig. 2. At each time, the mediators have a cross-sectional effect on the response, $M_1$ affects $Y_2$ and $Y_1$ affects $M_2$. Moreover, both the mediator and the outcome have autoregressive effects.

If the model in Fig. 2 reflects the true data generating mechanism, then assumptions **A0**-**A2** are satisfied. As a consequence, applying Eq. (1) , $\mathbb {E}[Y_2(X^Y = x, X^M= x^{\prime} )]$ results identified by

$$\begin{aligned} \sum _{m_1,m_2,y_1} \mathbb {E}[Y_2 \, | \, m_1, m_2, y_1, x ] P(m_2|m_1,y_1,x^{\prime })P(y_1\,| \,m_1,x)P(m_1|x^{\prime }). \end{aligned}$$

In the next sections, we apply the separable effects approach to mixed effects and latent growth models and derive analytical expressions for the mediational effects.

## Mixed effect models

Mixed effect models, also known as multilevel or hierarchical models, have traditionally been used for nested data, where observations belong to different groups or clusters. Examples of nested data are children in schools, people living in different areas of a city, or patients on which repeated measurements are taken over time. This class of models assumes the presence of random components which encode individual deviations from the population average. By using [17] notation, a mixed effect model can be specified as

2 $$\begin{aligned} Y_{ij} = \textbf{X}_{ij}^{ T} \varvec{\beta } +\textbf{Z}_{ij}^{T} \textbf{u}_j+ \varepsilon _{ij} \end{aligned}$$

where *i* and *j* denote the subject and the cluster, respectively, $Y_{ij}$ is the response variable for subject *i* in cluster *j*, $\textbf{X}_{ij}$ and $\textbf{Z}_{ij}$ are $p\times 1$ and $q \times 1$ vectors of known covariates, $\varvec{\beta }$ and $\textbf{u}$ are $p\times 1$ and $q \times 1$ vectors of fixed and random coefficients, respectively, with $\mathbb {E}[\textbf{u}] = 0$ and $\varepsilon _{ij}$ an error term with null expectation. The variables in $\textbf{Z}_{ij}$ are generally a subset of those in $\textbf{X}_{ij}$.

In a longitudinal mediation setting, mixed effect models could be fitted for both the mediator and the outcome, so that for each subject *i*

3 $$\begin{aligned} M_{it} = \textbf{X}_{Mit}^T \, \varvec{\beta } + \textbf{Z}_{Mit}^T \textbf{b}_{i} + \varepsilon _{Mit} \end{aligned}$$

4 $$\begin{aligned} Y_{it} = \textbf{X}_{Yit}^T \, \varvec{\gamma } + \textbf{Z}_{Yit}^T \textbf{g}_{i} + \varepsilon _{Yit}. \end{aligned}$$

In the simplest case $\textbf{X}_{Mit}$ includes only the intercept and the exposure and the same for $\textbf{X}_{Yit}$ with the addition of the mediator at time *t*. The vectors $\varvec{\beta }$ and $\varvec{\gamma }$ are fixed effects common to all subjects, while $\textbf{b}$ and $\textbf{g}$ are subject-specific random effects.

**Fig. 3:**
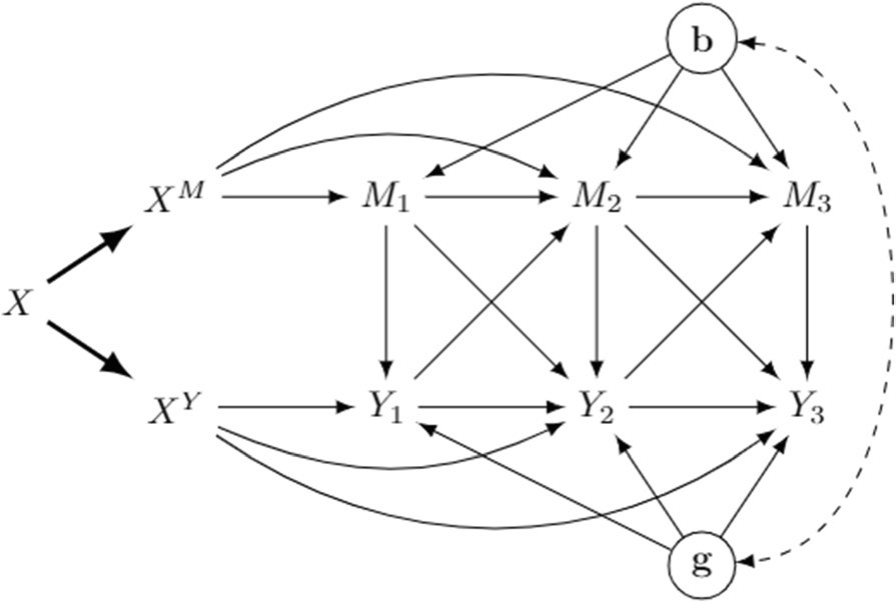
Three-wave mixed effect model with separable components of *X*. **b** and **g** are random effects

We have mentioned work dealing with longitudinal mediation using causal mixed-effect models. Among them, [5] propose assumptions for the identifiability of natural mediational effects in a simple setting without time-varying confounding. We consider a causal structure similar to theirs, but slightly modified by considering a baseline exposure *X* and a mediator and a response measured at different time occasions. Unlike [5], we allow the mediators and the outcome to be directly linked, not just through the random coefficients in the models. In addition, cross-lagged effects are allowed. These changes make the mediator and the outcome also time-varying confounders. Figure 3 shows the data structure for three waves.

We shall interpret the DAG in Fig. 3 as in the structural equation framework introduced by [33] (notice that we have explicitly included random effects as nodes in the graph). However, in contrast to the natural mediational effects, we do not consider any intervention on the mediators, but only on the separate components of the exposure.

The graph encodes some dependencies among variables: first, notice that the only children of $X^M$ are the mediators, and the only children of $X^Y$ are the outcome measurements over time. This implies that $X^M$ is independent of the outcome conditional on the mediators, and $X^Y$ is independent of the mediators conditional on the outcomes and previous mediators. Second, notice that the bidirected arrow connecting random effects is dashed, meaning that they can be marginally independent or correlated. Whether this arrow is present or not has dramatic impacts on the identifiability of separable effects. We will address the case of uncorrelated and correlated random effects in turn.

### Uncorrelated random effects

Let us start from the easiest case, where the two sets of random effects are uncorrelated, i.e. there is no dashed link in Fig. 3. In such a model, assumptions **A1** and **A2** ensure identifiability of the separable mediational effects. To show this, we start by considering the interventional expectation of the outcome under an intervention setting $X^M = x^{\prime}$ and $X^Y= x,$ with $x, x^{\prime} \in \{0,1\}, \, x \not = x^{\prime}$. Applying the law of iterated expectation:

5 $$\begin{aligned} & \mathbb {E}[Y_t( X^Y = x, X^M =x^{\prime} )] = \nonumber \\ & \sum _{\overline{m}_t, \overline{y}_{t-1}} \mathbb {E}[Y_t( X^Y = x, X^M =x^{\prime} ) \, | \, \overline{M}_t(X^Y = x, X^M =x^{\prime} ) = \overline{m}_t, \, \nonumber \\ & \qquad \qquad \qquad \qquad \qquad \qquad \qquad \qquad \quad \overline{Y}_{t-1}(X^Y = x, X^M =x^{\prime} ) = \overline{y}_{t-1}] \times \nonumber \\ & \prod _{k=1}^t P(M_k( X^Y = x, X^M =x^{\prime} ) = m_k \, | \,\, \overline{M}_{k-1}( X^Y = x, X^M =x^{\prime} ) = \overline{m}_{k-1}, \, \nonumber \\ & \qquad \qquad \qquad \qquad \qquad \qquad \qquad \qquad \quad \overline{Y}_{k-1}( X^Y = x, X^M =x^{\prime} ) = \overline{y}_{k-1}) \times \nonumber \\ & P(Y_{k-1}( X^Y = x, X^M =x^{\prime} ) = y_{k-1} \, | \, \overline{M}_{k-1}(X^Y = x, X^M =x^{\prime} ) = \overline{m}_{k-1}, \, \nonumber \\ & \qquad \qquad \qquad \qquad \qquad \qquad \qquad \qquad \quad \overline{Y}_{k-2}( X^Y = x, X^M =x^{\prime} ) = \overline{y}_{k-2}) \end{aligned}$$

with the assumption that variables with zero or negative subscripts are not present.

From assumption **A1** and Property **P1** it follows that, for each *k*,

$$\begin{aligned} & P(M_k(X^Y = x, X^M =x^{\prime} ) = m_k\, | \, \overline{M}_{k-1}(X^Y = x, X^M =x^{\prime} ),\nonumber \\ & \qquad \qquad \qquad \qquad \qquad \qquad \qquad \qquad \qquad \qquad \overline{Y}_{k-1}(X^Y = x, X^M =x^{\prime} ) )\nonumber \\ & \qquad \quad = P(M_k(X^Y = x^{\prime} , X^M =x^{\prime} ) = m_k\, | \, \overline{M}_{k-1}(X^Y = x^{\prime} , \, X^M = x^{\prime} ),\nonumber \\ & \qquad \qquad \qquad \qquad \qquad \qquad \qquad \qquad \qquad \qquad \overline{Y}_{k-1}(X^Y = x^{\prime} , \, X^M = x^{\prime} ) )\nonumber \\ & \qquad \quad = P(M_k(X = x^{\prime} ) = m_k\, | \, \overline{M}_{k-1}(X = x^{\prime} ), \overline{Y}_{k-1}(X = x^{\prime} ) ) \end{aligned}$$

and, since the treatment is randomised, this equals

$$\begin{aligned} P(M_k = m_k\, | \, X = x^{\prime} , \overline{M}_{k-1}, \overline{Y}_{k-1} ). \end{aligned}$$

The same holds for $Y_k$ by using **A2** instead of **A1**, i.e.

$$\begin{aligned} P(Y_k(X^Y = x, \, X^M = x^{\prime} ) & = y_k\, | \, \overline{M}_{k}(X^Y = x, \, X^M = x^{\prime} ), \overline{Y}_{k-1}( X^Y = x, \, X^M = x^{\prime} ) ) \\ & = P(Y_k(X^Y =x, X^M = x) = y_k\, | \, \overline{M}_{k}( X^Y =x, X^M = x), \\ & \kern8.5cm \overline{Y}_{k-1}( X^Y =x, X^M = x) ) \\ & = P(Y_k(X = x) = y_k\, | \, \overline{M}_{k}(X = x), \overline{Y}_{k-1}(X=x) ) \\ & = P(Y_k = y_k\, | \, X = x, \overline{M}_{k}, \overline{Y}_{k-1} ). \end{aligned}$$

It then follows that

6 $$\begin{aligned} \mathbb {E}[Y_t(X^Y = x, \, X^M = x^{\prime} )] = & \nonumber \\ & \sum \limits _{\overline{m}_t, \overline{y}_{t-1}} \mathbb {E}[Y_t\, |\, X = x, \, \overline{M}_t = \overline{m}_t, \, \overline{Y}_{t-1} = \overline{y}_{t-1}] \times \nonumber \\ & \prod _{k=1}^t P(M_k = m_k \, | \, X = x^{\prime} ,\, \overline{M}_{k-1} = \overline{m}_{k-1}, \, \overline{Y}_{k-1} = \overline{y}_{k-1}) \times \nonumber \\ & P( Y_{k-1} = y_{k-1} \, | \, X = x, \, \overline{M}_{k-1} = \overline{m}_{k-1}, \, \overline{Y}_{k-2} = \overline{y}_{k-2}). \end{aligned}$$

Specifying parametric models for the mediator and the outcome allows us to derive separable direct and indirect effects in terms of regression coefficients. For example, assume a structure as shown in Fig. 3. For each subject $i = 1, \dots , \, n$ and time occasion $t = 1, \dots , \, T$, if the mediator and the outcome are assumed to be Normally distributed and their expectations to be linear in the direct causes, possible models for their expectations can be

7 $$\begin{aligned} \mathbb{E}[M_{it} \, | \, X_i, \, \overline{M}_{it-1},\, \overline{Y}_{it-1}, \textbf{b}_i] = (\beta_0 + b_{0i}) + \beta_{X} X_i + \beta_{{\ell_1(M)}} M_{it-1} + \beta_{{\ell_1(Y)}} Y_{it-1} \end{aligned}$$

8 $$\begin{aligned} &\mathbb{E}[Y_{it} \, | \, X_i, \, \overline{M}_{it},\, \overline{Y}_{it-1},\textbf{g}_i] = \nonumber \\ & (\gamma_{0} + g_{{0i}}) + \gamma_{X} X_i + (\gamma_{{M_{t}}} + g_{{M i}}) M_{it} + (\gamma_{{\ell_1(M)}} + g_{{\ell_1(M)i}}) M_{it-1} + \gamma_{{\ell_1(Y)}} Y_{it-1}. \end{aligned}$$

where the subscripts $\ell _1(M)$ and $\ell _1(Y)$ denote the coefficients referring to $M_{t-1}$ and $Y_{t-1}$ respectively ($\ell _1$ stands for the lag operator of order 1), and we are assuming that

$$\begin{aligned} \mathbf{u}_i = (b_{0i}, \, g_{0i}, \, g_{{M i}}, \, g_{{\ell_1(M)i}} )^{\prime} \sim \text {MVN}(\varvec{0}, \varvec{\Phi }), \end{aligned}$$

with $$\varvec{\Phi }$$ diagonal.

Going back to the initial example of breastfeeding and weight, these models imply that the being breastfed has an effect both on weight and sugar craving over time. Subjects can show heterogeneity in the extent to which the mediator affects the response, so that the effect of $M_t$ and $M_{t-1}$ on weight at time *t* may vary across subjects. The random effects $g_{{M i}}$ and $g_{{\ell _1(M)i}}$ are included in the model to capture such heterogeneity. It is also plausible that the mediator and the outcome have autoregressive as well as cross-lagged effects, since, for example, a higher sugar craving at time *t* may lead to an increased weight at the subsequent measurement.

Suppose that one is interested in the separable effects of the exposure on the outcome at time $t = 2$. Considering the difference as contrast and two different values of $X, \, x$ and $x^{\prime}$, it can easily be proved that, applying the g-formula in (6), the separable effects, conditional on random effects, take the form

9 $$\begin{aligned} SDE_{|\mathbf{b,g}} = \gamma_{{X}} \big [ \, 1 + \beta_{{\ell_1(M)}} (\gamma_{{M}} + g_{{Mi}}) + \gamma_{{\ell_1(Y)}} \, \big ] (x-x^{\prime}\,) \end{aligned}$$

and

10 $$\begin{aligned} SIE_{|\mathbf{b,g}} = \,& \beta_{X} \big [ \, (\gamma_{{M}} + g_{{Mi}}) + \beta_{{\ell_1(M)}} (\gamma_{{M}} + g_{{Mi}}) + (\gamma_{M} + g_{{Mi}})\beta_{{\ell_1(Y)}}(\gamma_{{M}} + g_{{Mi}}) \\ & + (\gamma_{{\ell_1(M)}} + g_{{\ell_1(M)i}}) + (\gamma_{{M}} + g_{{Mi}})\gamma_{{\ell_1(Y)}} \, \big ] (x-x^{\prime}). \end{aligned}$$

Since one is usually interested in the average causal effects, random effects in the formulas above can be integrated out. In addition, since the random effects are assumed to be uncorrelated, the resulting effects are obtained by simply deleting the random coefficients, i.e.

11 $$\begin{aligned} SDE = \gamma_{{X}} \big [ \, 1 + & \beta_{{\ell_1(M)}} \gamma_{{M}} + \gamma_{{\ell_1(Y)}} \, \big ] (x-x^{\prime}\,) \end{aligned}$$

12 $$\begin{aligned} SIE = \beta_{X} \big [ \, \gamma_{{M}} + \beta_{{\ell_1(M)}}\gamma_{{M}} & + \beta_{{\ell_1(Y)}}\gamma_{{M}}^2 + \gamma_{{\ell_1(M)}} + \gamma_{{M}} \gamma_{{\ell_1(Y)}} \, \big ] (x-x^{\prime}). \end{aligned}$$

It is interesting, but not entirely surprising, to notice that each term of these effects refers to a path contributing to the effect under examination: the SDE includes products of coefficients along all the paths connecting $X^Y$ to $Y_2$, that is $X^Y \rightarrow Y_2, \, X^Y \rightarrow Y_1 \rightarrow M_2 \rightarrow Y_2$ and $X^Y \rightarrow Y_1 \rightarrow Y_2$, while SIE includes all path coefficients between $X^M$ and $Y_2$, for instance the first two terms represent the paths $X^M \rightarrow M_2 \rightarrow Y_2$ and $X^M \rightarrow M_1 \rightarrow M_2 \rightarrow Y_2$. This means that the separable effects are functions of time, since the more time is elapsed between the baseline measurement and that of interest, the more paths are involved.

### Correlated random effects

The case of correlated random effects can be further divided into two sub-cases: the non-null correlation concerns random effects related to the same variable, i.e. $\varvec{\Phi }$ is block diagonal, $$\varvec{\Phi } = \left( \begin{array}{cc} \varvec{\Phi }_b & \varvec{0}\\ \varvec{0} & \varvec{\Phi }_g \end{array}\right) ,$$ or random effects are free to covary with any element, so that $\varvec{\Phi }$ is a full, non-diagonal matrix.

The former case is not conceptually different from that with uncorrelated effects, and assumptions **A1**-**A2** are still valid. The only difference is that integrating out random effects is less straightforward. To show how the correlation of random effects impacts the formulas of separable effects, let us consider again Normally distributed mediator and outcome, and models (7)-(8). Suppose that $g_{{Mi}}$ is correlated with $g_{{\ell _1(M)i}}$ in model (8), and the other random coefficients are uncorrelated.

In formulas (9)-(10) there are no paths involving both random terms, since the time elapsed is too short. But consider the separable indirect effect of *X* on $Y_3$: among the different paths contributing to this effect, there is $X^M \rightarrow M_1 \rightarrow Y_2 \rightarrow M_3 \rightarrow Y_3$, which is analytically expressed by the product $\beta_{X} (\gamma_{{\ell_1(M)}} + g_{{\ell_1(M)i}}) \beta_{{\ell_1(Y)}} (\gamma_{M} + g_{{Mi}})$. Given the correlation between $g_{M}$ and $g_{{\ell _1(M)}}$, random effects cannot simply be deleted as in the previous case.

To obtain an expression free of random terms, it is necessary to solve the integral

$$\begin{aligned} \iint (\gamma_{{\ell_1(M)}} + g_{{\ell_1(M)}}) (\gamma_{{M}} + g_{{M}}) \, f(g_{{\ell_1(M)}}, \,g_{{M}} ) \, dg_{{\ell_1(M)}} \, dg_{{M}} \end{aligned}$$

where *f* is the joint density of the two random factors. Since *f* is a bivariate Normal with zero mean and non-diagonal covariance matrix, it can be proved that the integral above reduces to $\gamma_{{\ell_1(M)}}\gamma_{{M}} + \phi_{{g\ell_1(M), gM}},$ with $\phi_{{g\ell_1(M), gM}} = \text{Cov}(g_{{\ell_1(M)}}, \, g_{{M}})$. Thus, the g-formula in (6) is not non-parametrically identified, since to derive the previous formula we assumed the Normality of random effects.

As the number of paths increases, the expressions for the separable effects become increasingly complex and deriving their closed form is not trivial. For this reason, if random effects are believed to be correlated and/or the mediator and the outcome models to be non-linear, one of the solutions is to implement a code for the g-formula, without trying to solve it analytically.

Let us move to the case of non-diagonal $\varvec{\Phi }$, so there is at least an element of **b** correlated to an element of **g**. In Fig. 3 the dashed bidirected arrow is then present. This simple modification makes separable effects unidentifiable, since the mediators and the outcomes are now part of the same unique district, which is recanting, since both treatment components affect nodes in the district [34]. In addition, assumptions **A1** and **A2** fail, since *M* is no longer conditionally independent from $X^Y$ and *Y* is not conditionally independent from $X^M$. These considerations shed light into the nature of districts characterizing mixed-effect models. Indeed, if random effects are uncorrelated, or if they are correlated only within their ‘block’, the mediators and the outcomes belong to two separate districts $\{ M_1, \dots , \, M_T\}$ and $\{Y_1, \dots , \, Y_T\}$, which are not recanting, since nodes in a district are affected by only one of the components of *X*. The link between separable components and recanting districts has already been noted by [26].

## Latent growth models

Latent growth curve models (LGMs) are commonly used in social and behavioural sciences to model individual trajectories of some variables of interest, like depression or reading abilities in children. They allow each observation in the sample to have its own growth parameters so as to capture individual differences in change over time [35].

Using [19] notation, consider *n* individuals for which a variable of interest *Y* has been measured at *T* different time occasions. For each $i = 1, \dots ,\, n$, and $t = 1, \dots ,\, T$, an LGM with two latent factors can be written as

13 $$\begin{aligned}Y_{it} = \eta_{{0i}} + \lambda_t \eta_{{1i}} + \varepsilon_{it},\end{aligned}$$

where $\eta _{{0i}}$ and $\eta _{{1i}}$ are a random intercept and slope, respectively, and $\lambda _t$ is a coefficient encoding time. The random factors can be modelled as the sum of common constant means $\mu _{\eta _0}$ and $\mu _{\eta _1}$ and stochastic fluctuations, i.e. $\eta_{{0i}} = \mu_{\eta_0} + \zeta_{\eta_{0}i}, \, \eta_{{1i}} = \mu_{\eta_1} + \zeta_{\eta_{1}i}$ (*unconditional* LGM), or they can depend on some additional covariates (*conditional* LGM). Usually, to ensure the identifiability of model parameters, some constraints are imposed, for example the errors of observed variables are assumed to be uncorrelated with those of latent variables, i.e. $\text {cov}(\varepsilon _{it}, \zeta _{\eta _0 i}) = \text {cov}(\varepsilon _{it}, \zeta _{\eta _1 i}) = 0$, the variances of observed variables may be assumed constant over time, or the time parameter $\lambda _t$ can be fixed instead of being estimated. The choice of time coding is crucial, since it determines the interpretation of the latent factors. One of the most common choices is to fix $\lambda _t = t-1, \, t=1, \dots , T$, which represents the assumption of linear change in one time lag. In this case, the latent intercept can be interpreted as the average of *Y* at the first time occasion, and the latent slope as the rate of change.

Cheong et al. [2] and [3] address mediation models in an LGM framework by separately modelling the mediator and the outcome process and making the latent coefficients of the outcome model depend on those of the mediator and on the exposure, as described by the following equations:

14 $$\begin{aligned} \textbf{M}_i = \mathbf {\Lambda }_M \, \varvec{\eta }_{Mi} + \varvec{\varepsilon }_{Mi} \end{aligned}$$

15 $$\begin{aligned} \textbf{Y}_i = \mathbf {\Lambda }_Y \, \varvec{\eta }_{Yi} + \varvec{\varepsilon }_{Yi} \end{aligned}$$

which are the mediator and the outcome process, respectively. $\textbf{M}_i$ and $\textbf{Y}_i$ are *T*-dimensional vectors of repeated measures for subject *i*, $\varvec{\eta }_{Mi}$ and $\varvec{\eta }_{Yi}$ are two-dimensional vectors containing the latent growth factors $\eta _{0i}$ and $\eta _{1i}$, $\mathbf {\Lambda }$ is a $T\times 2$ matrix of coefficients for the latent factors, usually the first column is a vector of 1, the second accounts for time, and finally $\varvec{\varepsilon }_{Mi}$ and $\varvec{\varepsilon }_{Yi}$ are individual error vectors. So an example of mediator model in its extended form may be

$$\begin{pmatrix}M_{i1}\\ M_{i2}\\ \vdots\\M_{iT}\end{pmatrix} = \left(\begin{array}{cc}1&0\\ 1&1\\ \vdots&\vdots\\ 1&T\end{array}\right)\begin{pmatrix} \eta_{0Mi}\\ \eta_{1Mi}\end{pmatrix} + \left(\begin{array}{c} \varepsilon_{Mi1}\\ \varepsilon_{Mi2}\\ \vdots\\ \varepsilon_{MiT}\end{array}\right).$$

The latent factors are linked through the following relationships

16 $$\begin{aligned} \eta _{{0Mi}} = \beta _{00} + \beta _{01} X_i + \zeta _{\eta_{0 M i}} \end{aligned}$$

17 $$\begin{aligned} \eta _{{1Mi}} = \beta _{10} + \beta _{11} X_i + \zeta _{\eta_{1 M i}} \end{aligned}$$

18 $$\begin{aligned} \eta _{{0Yi}} = \gamma _{00} + \gamma _{01} X_i + \zeta _{\eta_{0 Y i}} \end{aligned}$$

19 $$\begin{aligned} \eta _{{1Yi}} = \gamma _{10} + \gamma _{11} X_i + \gamma _{12}\eta _{{0Mi}} + \gamma _{13}\eta _{{1Mi}} + \zeta _{\eta_{ 1 Y i}}. \end{aligned}$$

**Fig. 4:**
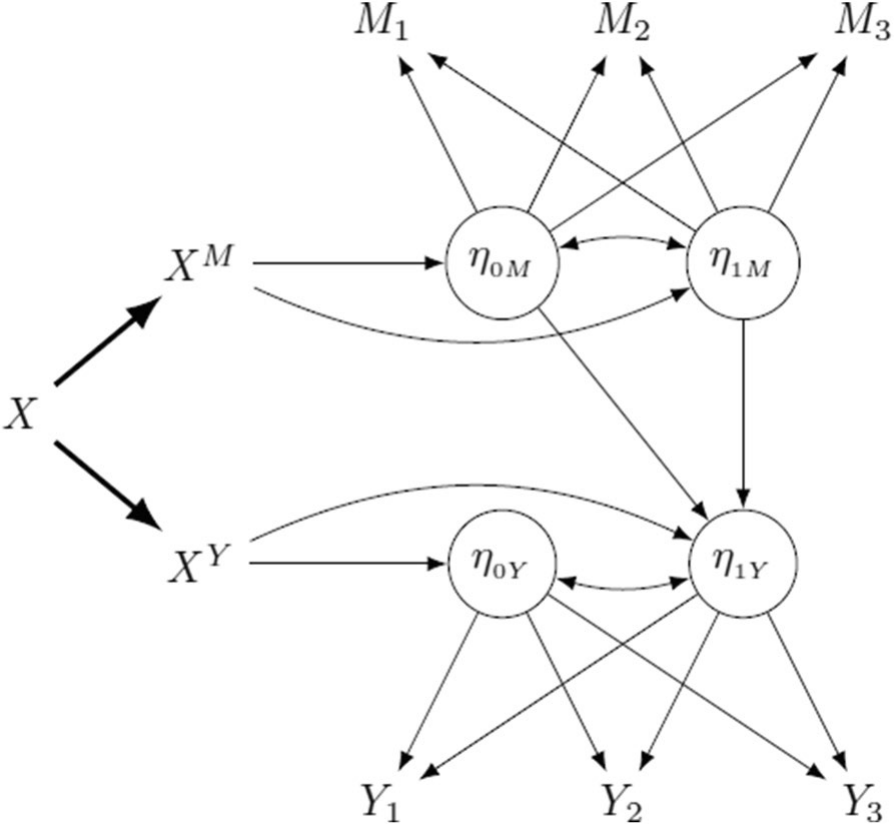
Three-wave latent growth model with separable components of *X*

All latent coefficients depend on the exposure, but, in addition, the outcome latent slope depends on the mediator latent growth factors. A graphical representation including separable components of *X* is given in Fig. 4. Clearly, models can be made (reasonably) more complex by adding non-linear components or other dependencies among latent factors.

Notice the differences between models (14) - (15) and the mixed effect models in Eqs. (3) - (4): in LGMs there is not a direct relationship between the observed variables, that is, the repeated measurements of mediator and outcome, instead they are indirectly connected through their latent factors. In mixed-effect models random effects explain heterogeneity among subjects and can be viewed as deviations from a common mean, while latent factors in LGMs determine the trajectories of observed variables over time. From another point of view, mixed effect models assume that the phenomenon happens at the level of repeated measures. In contrast, in LGMs, where a latent structure underlying the object of investigation is assumed, observed measurements are just indicators of this structure, since relationships of association and dependence involve random factors, not measurements.

The different specification impacts also the definition of intervention and the corresponding causal interpretation of effects. In mixed-effect models we assume that intervening on $X^M$ produces a change on the mediator and, likewise, an intervention on $X^Y$ modifies the outcome, at each time. In contrast, in LGMs, intervening on $X^M$ possibly leads to a change in the latent intercept $\eta _{{0M}}$ and the latent slope $\eta _{{0Y}}$ of the mediator model. The same holds for $X^Y$ and the latent factors in the outcome model. Then, intervening on $X^M$ and $X^Y$ affects indirectly the measurements of the mediator and the outcome, respectively. Once again, the effects of the intervention work at a latent level and have an indirect impact on the observed variables.

Considering again the running example, intervening on breastfeeding affects the mediator intercept, i.e. the average level of sugar craving at the beginning of the study, and its slope, that is, sugar craving change rate, as well as the average weight at time 0 and its change rate. In turn, the latent factors of sugar craving may have an effect on those related to weight. This mechanism is different from that described for the mixed effect model, since treatment assignment does not modify sugar craving or weight directly, but it affects their latent determinants. So, in a sense, the causal mechanism acts at a different, underlying level.

In contrast to what happens for mixed models with uncorrelated random effects, the latent growth model is never non-parametrically identified. Nonetheless, a modified version of assumptions **A1** -**A2** allows us to express the effects in terms of model parameters.

We assume that, for each latent factor $\eta$ in the model, $\eta (x) \perp \kern-.4cm \perp X$. In addition, the assumptions embedded in the model described by Eqs. (14)-(19) are

- **A1.1**$_{LGM}$ The mediator latent factors are independent of $X^Y$ given $X^M$: $\eta _{{0M}} \perp \kern-.4cm \perp X^Y \, | \, X^M,\ \eta _{{1M}} \perp \kern-.4cm \perp X^Y \, | \, X^M$  
- **A1.2**$_{LGM}$ The outcome latent factors are independent of $X^M$ given $X^Y$ and the mediator latent factors: $\eta _{{0Y}} \perp \kern-.4cm \perp X^M \, | \, (X^Y, \eta _{{0M}}, \eta _{{1M}}), \; \eta _{{1Y}} \perp \kern-.4cm \perp X^M \, | \, (X^Y, \eta _{{0M}}, \eta _{{1M}}, \eta _{{0Y}})$  
- **A2**$_{LGM}$ For each time *t* the response $Y_t$ is independent of the value of $X^M$ conditional on all latent factors and $X^Y$, $Y_t \perp \kern-.4cm \perp X^M \, | \, (\eta _{{0M}}, \eta _{{1M}}, \eta _{{0Y}}, \eta _{{1Y}},\, X^Y)$

Applying again the law of iterated expectation one can derive expressions for the mediational effects as follows:

20 $$\begin{aligned} \mathbb {E} & [Y_t(X^Y = x, \, X^M =x^{\prime} )] = \nonumber \\ & \int \mathbb {E} \big [Y_t(X^Y = x, \, X^M =x^{\prime} ) \, | \, \eta _{{0M}}(X^Y = x, \, X^M =x^{\prime} ) , \eta _{{1M}}(X^Y = x, \, X^M =x^{\prime} ), \nonumber \\ & \eta _{{0Y}}(X^Y = x, \, X^M =x^{\prime} ), \eta _{{1Y}}(X^Y = x, \, X^M =x^{\prime} ) \big ] \times \nonumber \\ & f \big (\eta _{{0M}}(X^Y = x, \, X^M =x^{\prime} ) \big ) \, f \big (\eta _{{1M}}(X^Y = x, \, X^M =x^{\prime} ) \big ) \, \times \nonumber \\ & f \big (\eta _{{0Y}}(X^Y = x, \, X^M =x^{\prime} ) \big ) \, \times \nonumber \\ & f \big (\eta _{{1Y}}(X^Y = x, \, X^M =x^{\prime} )\,|\, \eta _{{0M}}(X^Y = x, \, X^M =x^{\prime} ), \nonumber \\ & \eta _{{1M}}(X^Y = x, \, X^M =x^{\prime} ), \, \eta _{{0Y}}(X^Y = x, \, X^M =x^{\prime} ) \big ) d\eta \end{aligned}$$

where by $d\eta$ we mean that the integral is with respect to all the latent variables, and $f(\cdot )$ is the density of the latent factors; they are generally assumed to have a multivariate Normal distribution. Making use of property **P1** and assumptions **A0**, **A1.1**$_{LGM}$, **A1.2**$_{LGM}$ and **A2**$_{LGM}$ yields

21 $$\begin{aligned} \mathbb {E} & [Y_t(X^Y = x, \, X^M =x^{\prime} )] =\nonumber \\ & \qquad \qquad \qquad \qquad \int \mathbb {E} [Y_t\, |\,X = x, \, \eta _{{0M}} , \, \eta _{{1M}} , \eta _{{0Y}}, \eta _{{1Y}} ] f(\eta _{{0M}} \, | \, X = x^{\prime} ) \, \nonumber \\ & \qquad \qquad \qquad \qquad f(\eta _{{1M}} \, | \, X = x^{\prime} ) \,\, f(\eta _{{0Y}}\, | \, X = x) \, f(\eta _{{1Y}} \, | \, X = x, \, \eta _{{0M}} , \, \eta _{{1M}}, \, \eta _{{0Y}}) d\eta \end{aligned}$$

Looking at Eq. (21), it can be noted that it depends on both observed and unobserved variables and involves quantities non-identifiable in the absence of other parametric assumptions. Then, assumptions **A1.1**$_{{LGM}}$, **A1.2**$_{{LGM}}$ and **A2**$_{{LGM}}$ are useful for expressing the interventional expectation $\mathbb {E} [Y_t(X^Y = x, \, X^M =x^{\prime} )]$ as in (21). Parametric identification is achieved by exploiting the parametric assumptions encoded by LGMs about the distribution of the latents.

For a model like that described in Eqs. (14)-(19), the separable direct effect on the difference scale is

22 $$\begin{aligned} SDE = (\gamma _{01} + \gamma _{11} \lambda _t)(x-x^{\prime} ) \end{aligned}$$

and the separable indirect effect

23 $$\begin{aligned} SIE = \lambda _t(\beta _{01} \gamma _{12} + \beta _{11}\gamma _{13})(x-x^{\prime} ). \end{aligned}$$

It is easy to notice that these expressions are the same ones that would be obtained by means of path analysis, since the effects are defined as sums of path-specific effects, which are in turn obtained as product of coefficients lying on the path. For example, the direct effect is obtained by summing the effect through the path $X^Y \rightarrow \eta _{{0Y}} \rightarrow Y_t$ and that through $X^Y \rightarrow \eta _{{1Y}} \rightarrow Y_t \,\,\, \forall \, t$. Moreover, Eqs. (22)-(23) are consistent with the expressions for natural effects obtained by [23]. However, the separable effects approach is much more intuitive, since it does not require any intervention on the model latent variables and the assumptions are all ‘single-world’, verifiable from the graph directly.

As remarked for mixed effect models, also, in this case, the mediational effects are time-varying. Notice, however, that Eqs. (9)-(10) are very different from (22)-(23). The former show time dependence in the fact that, at each *t*, the number of paths connecting variables, and thus the number of coefficients, increases. The latter encode time dependence only via $\lambda _t, \, t =1, \dots , \, T$.

## Simulation study

In this section, we conduct a simulation study to assess how the choice of an incorrect latent structure, either in the mediator or the outcome model, affect the estimation of separable effects via the g-formula. This may be due to model misspecification, or to the fact that identifying assumptions are not satisfied. We consider both cases and a simple scenario consisting of a binary exposure and Normally-distributed mediator and outcome for $T=5$ measurement occasions. Data were simulated from two different models: a linear mixed model as specified in Eqs. (7)-(8) with uncorrelated random effects, drawn from a multivariate Normal distribution with zero mean vector and identity covariance matrix, and a latent growth model as in Eqs. (16)-(19), where the $\zeta$ terms follow a standard Normal distribution. The model coefficients are reported in Table 1.

**Table 1:** Coefficients of the mixed-effect and latent growth models used to generate data

Mixed-effect model		LGM	
Coefficient	Value	Coefficient	Value
$\beta _{0}$	1.3	$\beta _{00}$	0.21
$\beta _{X}$	0.5	$\beta _{01}$	0.16
$\beta _{{\ell _1(M)}}$	0.27	$\beta _{10}$	0.7
$\beta _{{\ell _1(Y)}}$	0.11	$\beta _{11}$	0.47
$\gamma _{0}$	0.45	$\gamma _{00}$	0.3
$\gamma _{X}$	0.7	$\gamma _{01}$	0.14
$\gamma _{M{_t}}$	0.2	$\gamma _{10}$	0.59
$\gamma _{{\ell _1(M)}}$	0.08	$\gamma _{11}$	0.27
$\gamma _{{\ell _1(Y)}}$	0.34	$\gamma _{12}$	0.44
		$\gamma _{13}$	0.19

### Model misspecification

In principle, the g-formula does not require any parametric assumption. However, if the number of variables is large, it can be difficult to apply it without recurring to (semi-)parametric models. The parametric g-formula relies on the correct specification of such models. In general, researchers are concerned about ignoring relevant variables, i.e. unobserved confounding. In longitudinal settings, there is also the issue of modelling the dynamic aspect of the phenomenon, which can be done in a wide variety of ways, involving latent structures or not. When a latent structure is taken into account, it is, however, difficult to select the most appropriate one.

To analyse the extent to which estimates are affected by the use of wrong models, we considered two degrees of misspecification: moderate misspecification, where the model is very similar to the true one, except for a term, which is missing, and severe misspecification, where the models are completely wrong. Specifically, for the mixed-effect model we considered a moderate misspecification in the mediator model, which is assumed to be as in Eq. (7) but with $\beta _{{\ell _1(Y)}} = 0$; for the LGM, we did not include $\eta _{{0M}}$ in the model for $\eta _{{1Y}}$. As regards severe misspecification, the mixed-effect model was addressed as an LGM and, vice versa, the LGM as a mixed-effect model. This mirrors the case in which a researcher is completely agnostic about the true nature of the phenomenon under study.

For each generating mechanism, we simulated $K =500$ datasets with $n=1,000$ observations, on which we fitted each misspecified model and estimated the separable direct and indirect effects on the difference scale through the mediational g-formula. True values of the parameters were obtained straightforwardly for LGMs by applying formulas (22)-(23), while they were estimated asymptotically for mixed-effect models, since the analytical form of effects is more complex. We evaluated the relative bias defined as

$$\begin{aligned} \text {Relative bias} = \frac{\sum _{k=1}^K (\hat{\theta }_{kt} - \theta _t)}{K \theta _t}, \end{aligned}$$

where $\hat{\theta }_{kt}$ is the estimate of the SDE or the SIE at time *t* obtained in the *k*-th simulation; the root mean square error (RMSE)

$$\begin{aligned} RMSE = \sqrt{\frac{\sum _{k=1}^K (\hat{\theta }_{kt} - \theta _t)^2}{K}}, \end{aligned}$$

and the coverage rate of 95% confidence intervals, obtained through $B = 500$ bootstrap samples.

The g-formula algorithm can be divided into three steps:

1. For each $t=1,\dots , T$, fit parametric models for the mediator and the outcome conditional on the treatment and their histories, i.e. estimate densities $f_M(M_t | X, \overline{M}_{t-1}, \overline{Y}_{t-1})$ and $f_Y(Y_t | X, \overline{M}_{t}, \overline{Y}_{t-1})$.
2. Select $$S\ge 10,000$$.
  Specify an intervention or a set of interventions to compare, creating two variables $X^M$ and $X^Y$ and setting them to the values of interest.
  For each $s = 1, \dots , S$ and $t=1, \dots , T$, draw a value $\tilde{m}_{st}$ for the mediator from $f_M(M_t | x^M, \overline{\tilde{m}}_{st-1}, \overline{\tilde{y}}_{st-1})$ estimated in step 1, conditional on $x^M$ and its (simulated) history. Do the same for *Y*, conditional on $x^Y$ and its history, i.e. draw $\tilde{y}_{st}$ from $f_Y(Y_t | x^Y, \overline{\tilde{m}}_{st}, \overline{\tilde{y}}_{st-1})$. For continuous distributions whose variance is not a function of the mean, the variance is estimated through the model residual mean squared error [36].
3. Compute the intervention mean estimate at each time $t=1, \dots , T$, by averaging the outcome expectation over simulated subjects:
  24 $$\begin{aligned} \mathbb {E}[Y_t( X^Y = x^Y, X^M = x^M)] = \frac{1}{S} \sum _{s=1}^S \mathbb {E}[\tilde{y}_{st}] \end{aligned}$$

Standard errors and confidence intervals for the average intervention effect can be estimated through non-parametric bootstrap, by repeating steps 2 and 3 *B* times, where *B* is the number of bootstrap samples.

The separable direct and indirect effects can easily be obtained by comparing expressions of the form (24), appropriately selecting $x^M$ and $x^Y$. For example, if one wants to estimate the SDE and *X* is binary, one should compare $X^M = 0,\, X^Y = 1$ with $X^M = 0, \, X^Y = 0$.

### Results

Simulations were conducted in the statistical software R, version 4.2.0. Results are shown in Tables 2 and 3. As expected, for data generated from a mixed-effect model, moderate misspecification produced an underestimation of both SDE and SIE. This is consistent with the fact that the term expressing the lagged influence of the outcome was removed from the mediator model. When the misspecification is severe, the SDE is underestimated, while the SIE is overestimated, and the estimates are progressively farther from the true values as the amount of time elapsed increases. Both relative bias and RMSE are smaller (in absolute values) for effects estimated through the moderately misspecified model than for effects estimated using the severely misspecified model, and those of direct effects are generally lower than those of the indirect effects. In addition, they show an increasing trend over time. Coverage rates are lower than the nominal level and they decrease as time elapses, so they are higher for the effect of *X* on *Y* at the first time occasions and tend to become smaller at subsequent times. The SDE coverage rates are very similar in both cases of misspecification, while for SIE they appear higher in the severe case, except for time 1.

**Table 2:** Results of simulations for data generated from a mixed model as in Fig. 3 with uncorrelated random effects

Misspecification	True		Estimates		Rel. bias		RMSE		Coverage rate	
	SDE	SIE	SDE	SIE	SDE	SIE	SDE	SIE	SDE	SIE
Moderate	0.700	0.100	0.699	0.114	-0.001	0.140	0.100	0.029	0.866	0.832
	0.956	0.257	0.902	0.245	-0.056	-0.047	0.140	0.052	0.862	0.854
	1.064	0.384	0.961	0.316	-0.097	-0.178	0.173	0.096	0.784	0.668
	1.123	0.527	0.978	0.348	-0.129	-0.340	0.202	0.194	0.710	0.222
	1.162	0.660	0.983	0.361	-0.154	-0.453	0.229	0.309	0.622	0.320
Severe	-	-	0.880	0.000	0.257	-0.999	0.251	0.100	0.814	0.000
	-	-	0.796	0.355	-0.167	0.383	0.261	0.137	0.886	0.868
	-	-	0.712	0.711	-0.331	0.849	0.448	0.378	0.738	0.632
	-	-	0.628	1.066	-0.440	1.023	0.613	0.610	0.690	0.542
	-	-	0.544	1.422	-0.532	1.154	0.769	0.851	0.696	0.486

For every model, each row refers to a different time $t=1,\dots ,5$

**Table 3:** Results of simulations for data generated from a latent growth model as in Eqs. (14)-(15)

Misspecification	True		Estimates		Rel. bias		RMSE		Coverage rate	
	SDE	SIE	SDE	SIE	SDE	SIE	SDE	SIE	SDE	SIE
Moderate	0.140	0.000	0.138	0.000	-0.017	0.000	0.078	0.000	0.966	1.000
	0.410	0.160	0.479	0.089	0.169	-0.445	0.124	0.074	0.878	0.308
	0.680	0.319	0.821	0.177	0.207	-0.445	0.212	0.149	0.852	0.230
	0.950	0.479	1.162	0.266	0.223	-0.445	0.309	0.222	0.838	0.206
	1.220	0.639	1.504	0.354	0.233	-0.445	0.407	0.297	0.832	0.210
Severe	-	-	0.252	0.038	0.802	0.038	0.130	0.039	0.460	1.000
	-	-	0.456	0.112	0.031	-0.030	0.127	0.029	0.874	0.892
	-	-	0.625	0.329	-0.081	0.031	0.172	0.056	0.868	0.894
	-	-	0.770	0.546	-0.190	0.131	0.271	0.110	0.734	0.802
	-	-	0.897	0.794	-0.265	0.242	0.402	0.199	0.586	0.646

For every model, each row refers to a different time $t=1,\dots ,5$

Results for data generated from an LGM are less clear. The indirect effects estimated through the moderately misspecified model are smaller than the true ones, as expected, since the term $\beta _{01}\gamma _{12}$ in Eq. (23) is missing. Setting $\gamma _{12} = 0$ influences also the estimate of SDE, which, on the contrary, is overestimated. In contrast, in the situation with severe misspecification, the SDE is underestimated, while the SIE is overestimated. Compared to mixed-effect model, it seems that misspecification affects the estimates in a more severe way, even when misspecification is only modest.

Relative bias and RMSE do not show clear patterns, and, in fact, in most cases they are smaller in the severe misspecification condition. Coverage rates for SDE are higher in the case of moderate misspecification, although below the nominal level. For SIE, coverage rates are better in the severe case and this may be due to the fact that estimates are heterogeneous enough to include the true effects, while in the moderate misspecification case the estimates are too far from the true values and the confidence intervals are not wide enough to include them.

### Violation of assumptions

We run other simulations to assess how the violation of identifiability assumptions impacts the estimates of separable effects. We generated data from a mixed-effect and a latent growth model as described in the previous section, including also unobserved confounders violating some of the assumptions **A1**-**A2** and **A1.1**$_{{LGM}}$, **A1.2**$_{{LGM}}$ and **A2**$_{{LGM}}$, respectively. Specifically, in the mixed-effect model, we included an unobserved confounder between *M*_1_ and *Y*_1_ that violates assumption **A1**, since $Y_1$ is no longer independent of $X^M$, while in the LGM we included an unobserved confounder between $\eta _{{0M}}$ and *Y*, which makes the outcome depend on $X^M$, violating assumption **A2**$_{{LGM}}$. For each model, we generated data with a sample size of either 100 or 1000. Results are shown in Tables 4 and 5.

**Table 4:** Results of simulations for data generated from a mixed model as in Fig. 3 with uncorrelated random effects and an unobserved confounder between $M_1$ and $Y_1$

n	True		Estimates		Rel. bias		RMSE		Coverage rate	
	SDE	SIE	SDE	SIE	SDE	SIE	SDE	SIE	SDE	SIE
100	0.700	0.100	0.704	0.119	0.006	0.185	0.317	0.086	0.898	0.886
	0.956	0.257	0.907	0.250	-0.051	-0.029	0.410	0.173	0.888	0.862
	1.064	0.384	0.966	0.322	-0.092	-0.163	0.446	0.239	0.878	0.832
	1.123	0.527	0.984	0.355	-0.124	-0.326	0.464	0.311	0.862	0.732
	1.162	0.660	0.990	0.370	-0.149	-0.440	0.478	0.398	0.844	0.604
1000	-	-	0.697	0.122	-0.004	0.221	0.106	0.035	0.856	0.754
	-	-	0.899	0.255	-0.059	-0.006	0.148	0.053	0.816	0.860
	-	-	0.957	0.327	-0.101	-0.149	0.181	0.090	0.750	0.716
	-	-	0.974	0.359	-0.132	-0.318	0.211	0.185	0.674	0.282
	-	-	0.979	0.373	-0.158	-0.435	0.237	0.299	0.618	0.056

For every model, each row refers to a different time $t=1,\dots ,5$

**Table 5:** Results of simulations for data generated from an LGM as in Fig. 4 with an unobserved confounder between $\eta _{{0M}}$ and the outcome at all times. For every model, each row refers to a different time $t=1,\dots ,5$

n	True		Estimates		Rel. bias		RMSE		Coverage rate	
	SDE	SIE	SDE	SIE	SDE	SIE	SDE	SIE	SDE	SIE
100	0.140	0.000	0.124	0.000	-0.017	0.000	0.256	0.000	0.946	0.000
	0.410	0.160	0.399	0.163	-0.026	-0.019	0.340	0.178	0.958	0.966
	0.680	0.319	0.675	0.326	-0.007	-0.019	0.548	0.356	0.948	0.966
	0.950	0.479	0.951	0.488	0.001	-0.019	0.788	0.534	0.948	0.966
	1.220	0.639	1.227	0.651	0.006	-0.019	1.038	0.712	0.952	0.966
1000	-	-	0.140	0.000	0.002	0.000	0.079	0.000	0.938	0.000
	-	-	0.414	0.159	0.010	-0.003	0.098	0.041	0.944	0.964
	-	-	0.688	0.318	0.011	-0.003	0.152	0.082	0.948	0.964
	-	-	0.961	0.477	0.012	-0.003	0.217	0.122	0.960	0.964
	-	-	1.235	0.637	0.012	-0.003	0.284	0.163	0.958	0.964

For the mixed-effect model, the mediational effects are underestimated especially on later time occasions, and the reduction is more severe for the indirect effect. The RMSE and the coverage rates are obviously higher when $n=100$, although the coverage is always below the nominal level 0.95. Moreover, the coverage of SIE confidence intervals is lower than that of the SDE for the same time occasion.

For the LGM, the point estimates of mediational effects are not affected by the confounder, and also coverage rates are quite good. However, this is due to the fact that the estimates show a very high variability, especially in the scenario where $n=100$, where the confidence intervals always contain 0. When $n=1000$, it seems that the effect of the confounder is mitigated.

These results, although for relatively simple models, suggest that even a moderate misspecification can have a considerable impact on the estimates of separable effects, as well as on their confidence intervals. Thus, researchers should carefully choose the most appropriate and plausible latent structure and model specification, especially in a longitudinal setting, where the dynamics can be difficult to capture and many interactions among variables may be present. Although there are no ‘golden rules’ to choose an appropriate model, there are some data characteristics that may help. For example, focusing on the two models discussed in this paper, when data are balanced or time-structured, i.e. when each subject is measured at the same time occasions and the main interest lies in individual trajectories, LGMs are more appropriate. When instead one wants to assess the effect of several variables on the outcome, or the data are unstructured, mixed-effect models are a better choice. See Table 1 in [37] for a comparison, and [38, 39] for strategies to select the most appropriate mixed-effect model or LGM, respectively. In general, longitudinal data can be very complex, and a variety of statistical models have been proposed. Choosing among the numerous alternatives may be difficult, and it is not the main focus of this paper. Interested readers may want to consult [40, 41] for roadmaps to the choice and comparisons of different models.

## Data analysis

In this section, we consider the first four waves of data from the National Longitudinal Study of Adolescent to Adult Health (Add Health, [42]). The study’s first wave involved more than 90,000 US students in grades 7 through 12 during the 1994-1995 school year. Some of them were re-interviewed three times until 2008 and, at each wave, they were asked about different aspects of their lives and, at wave I, one of the parents, generally the mother, also provided information on the adolescent’s early childhood, family income, health insurance, and neighborhood. The subsequent questionnaires were administered in 1996, 2001 and 2008. The main aim of the study was to examine developmental and health trajectories across the life course of adolescence into young adulthood.

We used the public version of the data set, whose first wave includes more than 6,000 subjects and 1,000 variables. We selected only the subjects whose data were available at all waves so that the sample size was reduced to 1,659 individuals. Our interest lies in investigating the effect of breastfeeding on subjects’ body mass index (BMI) over time, measured in kg/m$^2$, with regular smoking as potential mediator, i.e. smoking at least one cigarette for thirty days. Both variables are time-varying, since they are measured at each wave. In terms of separable effects, the assumption is that breastfeeding has a metabolic component $X^Y$ (the intake of mother’s milk), acting on BMI directly, and another component $X^M$, making smoking uptake at an adult age less likely. This could be, for example, a neurological component affecting nicotine receptors. Previous studies highlighted the important role of breastfeeding in regulating adult weight and body fat [43, 44], while the relationship between risky or addictive behaviour in adulthood, like smoking and substance abuse, is yet to be investigated.

In more detail, the exposure (breastfeeding) is a categorical variable with seven levels, indicating how long each subject was breastfed. We used a dichotomised version of this variable, obtained by assigning value 1 to all the subjects breastfed for at least six months, 0 to the others. This threshold was selected on the basis of the World Health Organisation’s recommendations about breastfeeding. The mediator is a binary variable indicating if the subject smoked at least one cigarette a day in the thirty days before the interview. The outcome is continuous and positive. We modelled smoking as a binomial variable using a logit link and the outcome with a Gamma distribution with log link; the linear predictors are as in Eqs. (7)-(8), respectively. We also adjusted for several possible confounders, i.e. biological sex, age, weight at birth, average number of alcoholic drinks drunk in the last year, and whether the subject’s parents suffered from obesity, diabetes or alcoholism. The original data sets included hundreds of variables; therefore, the confounders to be included in the analysis were chosen using a two-step procedure. First, we selected a subset of more than thirty variables believed relevant based on subject matter knowledge (excluding, for example, variables like the number of the subject’s sexual partners or the times a subject was arrested). Under the assumption that this initial set of covariates is sufficient to control for confounding, we can use variable selection methods (here we used AIC) to reduce the set to those covariates that are actually relevant [45]. Results are shown in Table 6.

**Table 6:** Results of the analysis on the Add Health data, investigating the effects of breastfeeding on BMI through smoking. For each wave, estimates of SDE and SIE and the corresponding standard errors and confidence intervals are reported, based on 500 bootstrap replications

Wave	SDE	SIE	Prop. Mediated
1	-0.765 (0.126)	-0.011 (0.005)	0.014
	(-0.999, -0.516)	(-0.022, -0.002)	
2	-0.930 (0.153)	-0.030 (0.012)	0.031
	(-1.218, -0.626)	(-0.054, -0.008)	
3	-0.943 (0.155)	-0.032 (0.013)	0.033
	(-1.233, -0.639)	(-0.058, -0.007)	
4	-0.979 (0.162)	-0.032 (0.014)	0.032
	(-1.277, -0.658)	(-0.061, -0.006)	

The last column reports the proportion mediated, obtained as the ratio of the SIE and the total effect, SDE+SIE

It can be noticed that both separable effects are negative and significant at each wave and their magnitude (in absolute value) increases. This suggests that being breastfed for more than six months has a beneficial effect on BMI both directly and indirectly, by reducing the probability of smoking regularly which reduces BMI. The indirect effect is smaller than the direct one, with a proportion mediated (i.e. the ratio between the indirect effect and the total effect $\frac{SIE}{SDE+SIE}$) varying between 1 and 3% over the four waves. It is worth remarking that the confounders we included in the analysis are all time-fixed, except for the average number of alcoholic drinks the subject drank in the year previous to the interview. We assumed that this is not a post-treatment confounder, i.e. it is not affected by breastfeeding. Including it as a post-treatment confounder would have been possible (see [28], for example), but would have make the analysis more complex. Despite this limitation, our results provide an interesting starting point for future studies investigating the indirect effects of breastfeeding through its neurological component with a focus to risky/addictive behaviour in adulthood, like smoking and substance abuse.

## Conclusions

In this article, we have applied the separable effects approach for mediation analysis proposed by [24] to mixed-effect and latent growth models. For each of them, we proposed a set of assumptions which suffice for the identification of separable effects and derived formulas to estimate them using the g-formula.

When the relationships are linear, the separable effects can be expressed in closed forms that have a direct correspondence with the graphs representing the models. As we saw, the separable direct effect is the sum of effects along all paths starting from $X^Y$, while the separable indirect effect is obtained as the sum of all combinations of coefficients along paths having $X^M$ as starting node. This provides a connection with path analysis. Another advantage of the separable effects approach is that the estimands do not rely on the notion of intervening on the mediator: this feature is particularly useful in LGMs, where it would be difficult to conceive an intervention on the latent factors characterizing the mediator trajectory, as proposed in [23].

In our discussion, we focused primarily on linear models, although the g-formula in (6) can accommodate more complex models and variables with different distributions, as we showed in the application. However, the complexity of the models, in terms of non-linearity of link functions, interaction terms and order of lagged effects, impacts the estimation procedure in two ways: First, finding closed forms for mediational effects becomes unpractical, so they have to be obtained through simulation, as described in the Simulation study section. Second, the more complex the model, the higher the computational intensity of the algorithm. For example, our data analysis took around 30 minutes, while the simulation study took several hours, using an Intel core i7 PC with 16 GB of RAM. Especially for mixed-effect models, the computation time can become very long depending to the number of random effects included in the analysis. This is one of the drawbacks of the estimation via g-formula, mainly due to the need to use bootstrap for estimating the effects’ confidence intervals, a highly time-consuming task.

This issue can be exacerbated when the design is unbalanced. We have not addressed this complication, and we think that it could be an interesting extension of the present work. Unbalanced designs are the rule, not the exception in real-world analyses. We have considered balanced designs to make the presentation of the approach clearer and easier to follow, but we acknowledge that this is a limitation. A special case of unbalanced design is given by censoring in survival analyses, which has been addressed in a separable effect framework by [25, 28]. How design imbalances should be addressed in other model settings is yet to be investigated, although it should not add conceptual difficulties.

In our discussion, we focused on settings where the treatment is measured at baseline, but time-varying treatments are very common in epidemiology. To the best of our knowledge, this issue has not yet been addressed from a separable effects perspective, although [31] suggests a possible way to deal with it. The basic idea is to expand the model by including another component of the treatment which affects the treatment’s subsequent measurements. While this seems quite straightforward in the case of mixed-effect models, the presence of a time-varying treatment in LGMs is more difficult to address. Indeed, when also the treatment varies over time, its trajectory is modelled as well, by means of latent variables shaping the form of individual trajectories. In such a case, the separability of the treatment should involve its latent factors, thus for each of them we could have a latent separable component related to the mediator and another related to the outcome. Giving a meaningful interpretation to these latent components seems quite complex, as well as devising possible interventions on them. For these reasons, the separable effects approach for LGMs in settings with time-varying treatments seems to be inadvisable.

Finally, we want to remark that conceiving different and separate components of *X*, i.e. components having an effect only on the mediator or the outcome, respectively, and which can, in principle, be intervened upon separately, is not always feasible. When researchers believe that the outcome component exerts its effect also on the mediators or vice versa, the separable effects and perhaps causal mediation analysis altogether should be avoided as the practical relevance will be questionable.

## Data Availability

Data are freely available at https://doi.org/10.3886/ICPSR21600.v25. Harris, Kathleen Mullan, and Udry, J. Richard. National Longitudinal Study of Adolescent to Adult Health (Add Health), 1994-2018 [Public Use]. Carolina Population Center, University of North Carolina-Chapel Hill [distributor], Inter-university Consortium for Political and Social Research [distributor], 2022-08-09.

## Abbreviations

BMI: Body mass index
LGMs: Latent growth models
RMSE: Root mean squared error
SDE: Separable direct effect
SIE: Separable indirect effect
